# Stimulus-Dependent Regulation of Nuclear Ca^2+^ Signaling in Cardiomyocytes: A Role of Neuronal Calcium Sensor-1

**DOI:** 10.1371/journal.pone.0125050

**Published:** 2015-04-21

**Authors:** Shu Nakao, Shigeo Wakabayashi, Tomoe Y. Nakamura

**Affiliations:** 1 Department of Molecular Physiology, National Cerebral and Cardiovascular Center, Suita, Osaka, Japan; 2 Department of Cardiac Physiology, National Cerebral and Cardiovascular Center, Suita, Osaka, Japan; University of Newcastle, AUSTRALIA

## Abstract

In cardiomyocytes, intracellular calcium (Ca^2+^) transients are elicited by electrical and receptor stimulations, leading to muscle contraction and gene expression, respectively. Although such elevations of Ca^2+^levels ([Ca^2+^]) also occur in the nucleus, the precise mechanism of nuclear [Ca^2+^] regulation during different kinds of stimuli, and its relationship with cytoplasmic [Ca^2+^] regulation are not fully understood. To address these issues, we used a new region-specific fluorescent protein-based Ca^2+^ indicator, GECO, together with the conventional probe Fluo-4 AM. We confirmed that nuclear Ca^2+^ transients were elicited by both electrical and receptor stimulations in neonatal mouse ventricular myocytes. Kinetic analysis revealed that electrical stimulation-elicited nuclear Ca^2+^ transients are slower than cytoplasmic Ca^2+^ transients, and chelating cytoplasmic Ca^2+^ abolished nuclear Ca^2+^ transients, suggesting that nuclear Ca^2+^ are mainly derived from the cytoplasm during electrical stimulation. On the other hand, receptor stimulation such as with insulin-like growth factor-1 (IGF-1) preferentially increased nuclear [Ca^2+^] compared to cytoplasmic [Ca^2+^]. Experiments using inhibitors revealed that electrical and receptor stimulation-elicited Ca^2+^ transients were mainly mediated by ryanodine receptors and inositol 1,4,5-trisphosphate receptors (IP3Rs), respectively, suggesting different mechanisms for the two signals. Furthermore, IGF-1-elicited nuclear Ca^2+^ transient amplitude was significantly lower in myocytes lacking neuronal Ca^2+^ sensor-1 (NCS-1), a Ca^2+^ binding protein implicated in IP_3_R-mediated pathway in the heart. Moreover, IGF-1 strengthened the interaction between NCS-1 and IP_3_R. These results suggest a novel mechanism for receptor stimulation-induced nuclear [Ca^2+^] regulation mediated by IP3R and NCS-1 that may further fine-tune cardiac Ca^2+^ signal regulation.

## Introduction

Intracellular calcium (Ca^2+^) regulates various cellular functions. In the heart, it is essential for muscle contraction, which is regulated by excitation-contraction (E-C) coupling [1]. Electrical stimulation by the propagating action potential triggers Ca^2+^ influx through plasma membrane L-type Ca^2+^ channels. This activates ryanodine receptors (RyRs) to release Ca^2+^ from the sarcoplasmic reticulum (SR) into the cytoplasm, leading to muscle contraction. On the other hand, changes in local Ca^2+^ levels ([Ca^2+^]) also occur in subcellular compartments such as the nucleus [2]. In cardiomyocytes, nuclear [Ca^2+^] is increased by receptor stimulation with growth factors, endothelin-1, and angiotensin II. This Ca^2+^ elevation is regulated by another Ca^2+^ release channel, inositol 1,4,5-trisphosphate receptor (IP_3_R), through activation of phospholipase C, followed by IP_3_ generation [2–8]. IP_3_R-dependent nuclear Ca^2+^ signaling has a significant impact on cardiomyocyte gene transcription, which in turn can lead to cardiac hypertrophy [2,3].

IP_3_Rs are present on the nuclear envelope, an intracellular Ca^2+^ store, and they can directly release Ca^2+^ from the nuclear envelope into the nucleoplasm [3,9]. Therefore, regulation of nuclear [Ca^2+^] is suggested to be independent of cytoplasmic global [Ca^2+^] regulation. On the other hand, Ca^2+^ can also be propagated from the cytoplasm into the nucleoplasm through the nuclear pore complexes, the major gateway for ions and macromolecules. Thus, the precise mechanisms of nuclear [Ca^2+^] regulation and its relationship with cytoplasmic [Ca^2+^] regulation are still unclear. The following questions regarding [Ca^2+^] regulation under conditions of different stimuli such as electrical and receptor stimulations, have remained unanswered: 1) how are Ca^2+^ signals generated in the nucleus, and are the regulations autonomous; and 2) do distinct mechanisms exist for the nuclear [Ca^2+^] changes elicited by different stimuli such as electrical and receptor stimulations. Several studies have investigated nuclear and cytoplasmic [Ca^2+^] regulation in various types of cardiomyocytes [4–6,8,10]. All these experiments were performed using conventional fluorescent Ca^2+^ indicators (i.e., Fluo-3 AM, Fluo-4 AM, Asante calcium red AM, and Fura-2 AM), which have many advantages. However, they might leak fluorescence from one subcellular compartment to another (e.g., cytoplasmic fluorescence may overlap nuclear fluorescence). Therefore, it is important to use region-specific fluorescent Ca^2+^ indicators to measure the Ca^2+^ signals in subcellular compartments and compare the results with those obtained with conventional Ca^2+^ indicators.

In addition to these methodological issues, the molecules involved in nuclear Ca^2+^ regulation remain to be clarified. We had previously reported that IP_3_R-mediated cardiac hypertrophy is regulated by a Ca^2+^ binding protein, neuronal Ca^2+^ sensor-1 (NCS-1) [11]. NCS-1 is a small (~22 kDa) protein with four EF-hand motifs (three of which bind Ca^2+^), and it is N-terminally myristoylated [12]. NCS-1 is pivotal for various neuronal functions, including neurotransmitter release [13], synaptic plasticity [14,15], learning and memory [15–17], neurite growth [18], and neuronal survival [19]. However, aside from our own previous work, only a few groups have explored the role of NCS-1 in the heart [11,20]. Therefore, we were greatly interested in exploring whether or how this new Ca^2+^ regulator NCS-1 would modulate nuclear Ca^2+^ signals in the heart.

In the present study, we addressed two important issues: (1) how nuclear and cytoplasmic Ca^2+^ signals are distinctly triggered by electrical and receptor stimulations and (2) whether and how NCS-1 regulates these events, by using a recently developed genetically encoded fluorescent Ca^2+^ indicator GECO that can be expressed in specific subcellular sites such as the nucleus and cytosol ([21]), and the conventional fluorescent indicator Fluo-4 AM, under the same experimental conditions. On the basis of our results, we have proposed possible mechanisms for nuclear [Ca^2+^] regulation and its functional significance during electrical and receptor stimulations.

## Materials and Methods

### Animals

This study conforms to the National Institutes of Health guidelines (Guide for the Care and Use of Laboratory Animals). Animal care and experimental procedures followed the Animal Welfare Committee guidelines and were approved by the National Cerebral and Cardiovascular Center Research Institute (Permit Number: 14048). *Ncs1* ^*−/−*^ mice (C57BL/6-NCR) were generated and genotyped by polymerase chain reaction analysis of genomic DNA as well as by western blotting, as described previously [11]. Mice were anesthetized with isoflurane (1−2% for maintenance; 3% for induction) in oxygen from a precision vaporizer when they were used for experiments.

### Primary culture

Ventricular myocytes were isolated from 1- to 2-day-old mouse hearts and dissociated into single cells by trypsinization, as described previously [22]. After excluding non-myocytes by differential adhesion treatment, myocytes were seeded into collagen-coated glass-bottom dishes and incubated in Dulbecco’s modified Eagle’s medium supplemented with 10% fetal bovine serum.

### Adenoviral vector

Plasmids (G-GECO1, NLS-R-GECO, NLS-PV-DsRed, NES-PV-DsRed, and DsRed) were purchased from Addgene. Plasmids used to generate adenoviruses were constructed using Gateway Technology (Life Technologies, Carlsbad, CA), and the adenoviruses were obtained through standard procedures. The viral titers were measured using a QuickTiter Adenovirus Titer Immunoassay Kit (Cell Biolabs, Inc., San Diego, CA) and determined as 1.4 × 10^8^ plaque-forming units (pfu)/mL for NCS-1-HA, 2.0 × 10^9^ pfu/mL for G-GECO1, 7.3 × 10^8^ pfu/mL for NLS-R-GECO, 1.5 × 10^8^ pfu/mL for NLS-PV-DsRed, 2.5 × 10^8^ pfu/mL for NES-PV-DsRed, and 1.9 × 10^7^ pfu/mL for DsRed. Each adenovirus was used with a multiplicity of infection (MOI) of 50.

### Ca^2+^ imaging

Cultured neonatal mouse ventricular myocytes (NMVMs) were infected with adenoviruses encoding G-GECO1 and NLS-R-GECO, which target the cytoplasm and nucleus, respectively, and two days after infection, Ca^2+^ imaging experiments were performed. Experiments using Fluo-4 AM were performed as described previously [11]. Dishes were mounted on an inverted confocal microscope (Olympus 1X81, equipped with 60×/1.42 oil immersion objective lens) attached to a confocal laser-scanning unit (Olympus Fluoview FV1000; argon and krypton lasers). Myocytes were superfused with Ca^2+^-free (plus 1 mmol/L ethylene glycol tetraacetic acid) or Ca^2+^-containing modified Tyrode’s solution composed of 137 mmol/L NaCl, 5.4 mmol/L KCl, 1 mmol/L MgCl_2_, 1.8 mmol/L CaCl_2_, and 10 mmol/L *N*-(2-hydroxyethyl)-piperazine-*N*′-2-ethanesulfonic acid (HEPES; pH 7.4). The excitation and emission wavelengths were 488 nm and >510 nm for Fluo-4 AM and G-GECO1, respectively; and 543 nm and >600 nm for NLS-R-GECO and DsRed, respectively. Images were acquired using the FV10-ASW imaging software (Olympus Optical Co., Tokyo, Japan). Myocytes showing similar fluorescence intensities were selected for analysis. Cytosolic and nuclear [Ca^2+^] are expressed as the percentage of fluorescence intensity (*F*) relative to basal fluorescence (*F*_*0*_) in each region (a value stable for at least 5 min in resting conditions). The respective regions of interests (ROI) were monitored within the nucleus and cytoplasm of the same cell.

### Immunofluorescence

Cultured NMVMs were subjected to immunofluorescence analysis using standard procedures. Briefly, cells were fixed with 10% neutral buffered formaldehyde, permeabilized and blocked, and incubated with primary antibodies at 4°C overnight, followed by incubation with fluorescent-conjugated secondary antibodies. The cells were then observed under an inverted microscope (Olympus 1X81, equipped with 60×/1.42 oil immersion objective lens) attached to a confocal laser-scanning unit. Frozen sections obtained from mouse hearts were also subjected to a similar procedure without permeabilization. Images were acquired using the FV10-ASW imaging software at room temperature.

### Proximity Ligation Assay (PLA)

*In situ* PLAs were performed to quantify protein-protein interactions between NCS-1 and IP_3_Rs in cells using the Duolink kit (O-link Bioscience, Uppsala, Sweden). Using this protocol, only when the two target proteins are in close proximity (<40 nm), high concentration of fluorescence amplified from each interacted molecule becomes visible as a distinct bright dot when viewed with a fluorescence microscope. Briefly, cultured NMVMs were fixed, permeabilized, and blocked. The cells were then incubated with primary antibodies against hemagglutinin (HA; to detect of NCS-1-HA) and IP_3_RI-III, followed by the appropriate secondary antibodies containing unique DNA strands (called PLA probes). Anti-α-actinin antibody followed by Alexa Fluor 488-conjugated anti-mouse secondary antibody (Life Technologies) and 4′,6-diamidino-2-phenylindole (DAPI) solution (Dojindo, Kumamoto, Japan) were added to visualize the myocytes and nuclei, respectively. The samples were evaluated by confocal microscopy. Images were analyzed by the ImageJ software (NIH) using the Cell Counter plug-in to count the number of PLA signals for each α-actinin-positive cell.

### Antibodies and other materials

We used mouse monoclonal antibodies against NCS-1 (BD Biosciences, Franklin Lakes, NJ; 1:100 dilution), LAP2 (BD Biosciences; 1:500 dilution), SERCa2a (Sigma-Aldrich, St. Louis, MO; 1:200 dilution), and rat monoclonal antibody against HA (Roche, Basel, Switzerland; 1:500 dilution), as well as rabbit polyclonal IP_3_RI-III (Santa Cruz Biotechnology Inc., Dallas, TX; 1:100 dilution). 2-Aminoethoxydiphenyl borate, tetracaine, and human recombinant IGF-1 were purchased from Sigma-Aldrich. Xestospongin C was purchased from Wako Chemical Co. (Osaka, Japan).

### Statistical analysis

GraphPad Prism 4.0 was used for basic data analysis. Statistical comparisons were performed by unpaired Student’s *t* tests for 2-group-only analysis and by one-way analysis of variance (ANOVA) followed by Bonferroni-corrected *t* tests for multiple-group comparisons. A one-tailed *P*-value of <0.05 indicated statistical significance; group data are expressed as means ± standard error of the mean (SEM) or as representative traces.

## Results

### Effects of electrical stimulation on nuclear and cytoplasmic Ca^2+^ transients

**Fig 1:**
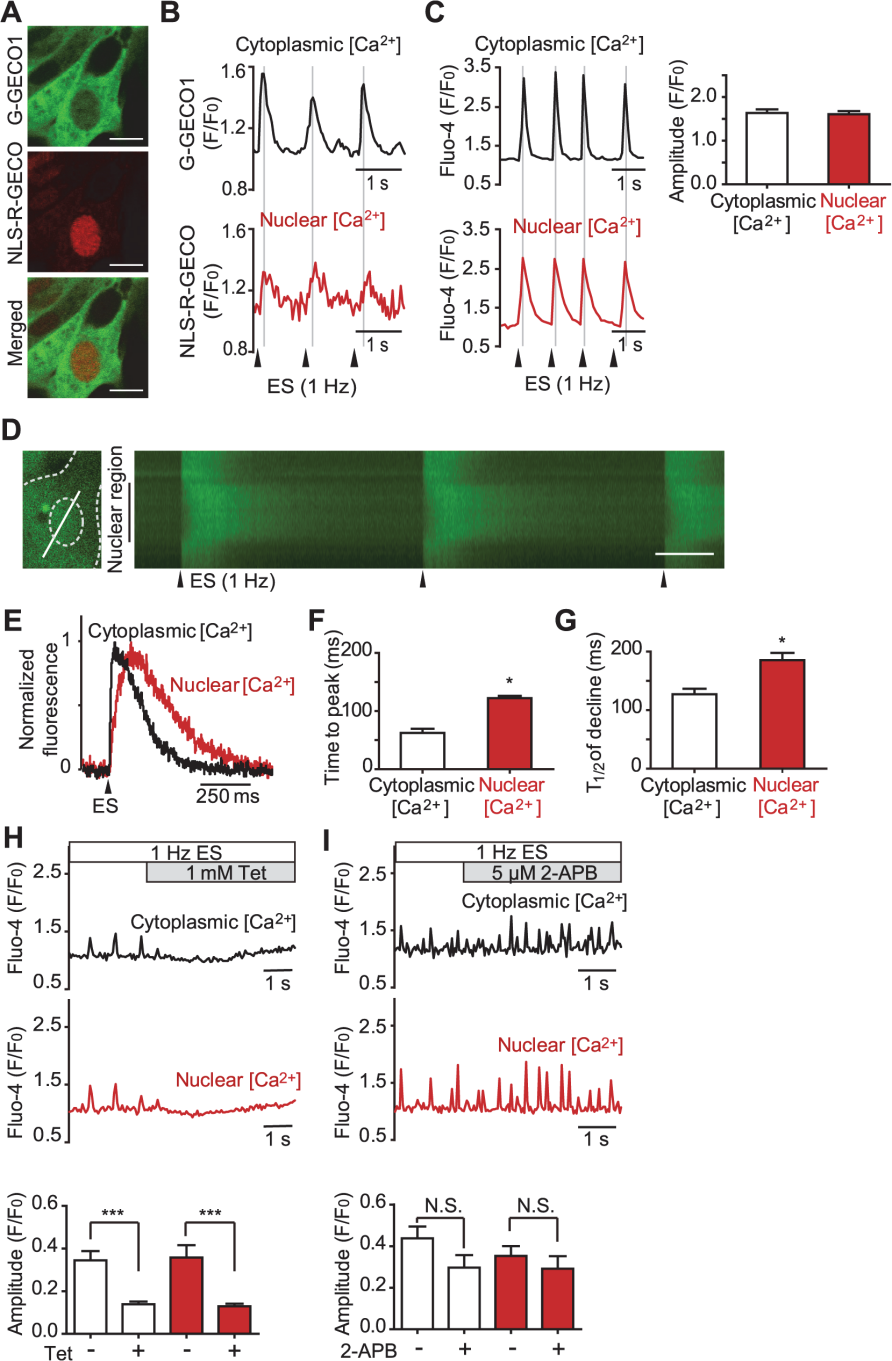
Cytoplasmic and nuclear Ca^2+^ levels in response to electrical stimulation in cardiomyocytes. (A) Representative images of cultured neonatal mouse ventricular myocytes (NMVMs) co-infected with adenoviruses encoding G-GECO1 and NLS-R-GECO. Bars, 10 μm. (B and C) Representative traces of cytoplasmic and nuclear Ca^2+^ transients elicited by 1-Hz electrical stimulation (ES) measured with GECOs (B) or Fluo-4 AM (C) as Ca^2+^ indicators. Each fluorescence intensity (F) was normalized to baseline fluorescence (F_0_) and presented as F/F_0_. The bar graph shows the averaged peak amplitude of cytoplasmic and nuclear Ca^2+^ transients in NMVMs loaded with Fluo-4 AM (n = 24 for each region). (D–G) Line-scan image analysis of electrical stimulation-elicited cytoplasmic and nuclear Ca^2+^ transients in NMVMs loaded with Fluo-4 AM. (D) Fluorescent image of the cell (left) and its line-scan image (right). White line in the cell image indicates the scan line including the cytoplasmic and nuclear region. White scale bar, 250 ms. (E) Normalized traces of the Ca^2+^ transients. (F and G) Averaged values of time to peak and decline time to half of the Ca^2+^ transients (n = 19). **P < 0.05 vs. cytoplasmic [Ca^2+^]. (H and I) Effect of inhibitors against ryanodine receptors (RyRs; 1 mmol/L tetracaine [Tet], n = 10, ***P < 0.001 vs. no inhibitor) (H) and inositol 1,4,5-trisphosphate receptors (IP_3_Rs; 5 μmol/L 2-aminoethoxydiphenyl borate [2-APB], n = 13) (I) on electrical stimulation-elicited Ca^2+^ transients. Values are expressed as means ± standard error of the mean (SEM).

We first examined the effects of electrical stimulation on cytoplasmic and nuclear [Ca^2+^] in cultured NMVMs using GECOs [21] as the Ca^2+^ indicator. We introduced green fluorescent G-GECO1 and red fluorescent NLS-R-GECO, which target the cytoplasm and nucleus, respectively, using adenoviral vectors, into NMVMs (Fig 1A). Electrical stimulation at 1 Hz elicited Ca^2+^ transients in both the cytoplasm and nucleus, and these Ca^2+^ transients were synchronized (Fig 1B). These results excluded the possibility of nuclear Ca^2+^ signals coming from the cytoplasm by fluorescent leakage. While these GECO probes are useful to monitor region-specific changes in [Ca^2+^], differences in their dynamic ranges preclude accurate comparison of cytoplasmic and nuclear Ca^2+^ transients. To address this issue, we performed similar experiments using a conventional Ca^2+^ indicator, Fluo-4 AM. Again, cytoplasmic and nuclear Ca^2+^ transients were synchronized with electrical stimulation, without significant changes in their amplitudes (Fig 1C). However, a more detailed analysis using the line-scan technique (Fig 1D) indicated that the kinetics of the nuclear Ca^2+^ transients were significantly slower than those of the cytoplasmic transients (Fig 1E), i.e. both time to peak and T_1/2_ of decline were significantly delayed (Fig 1F and 1G). These results suggest that electrical stimulation-elicited nuclear Ca^2+^ transients follow the dynamics of cytoplasmic [Ca^2+^]. To investigate which intracellular Ca^2+^ release channels are involved in the electrical stimulation-elicited nuclear Ca^2+^ transients, we examined the effects of various inhibitors. Tetracaine, an inhibitor of RyR, highly attenuated both cytoplasmic and nuclear Ca^2+^ transients, whereas 2-aminodeoxydiphenyl borate (2-APB), an inhibitor of IP_3_Rs, did not (Fig 1H and 1I), indicating that electrical stimulation-elicited nuclear Ca^2+^ transients are mediated by RyR, similar to cytoplasmic Ca^2+^ transients.

**Fig 2:**
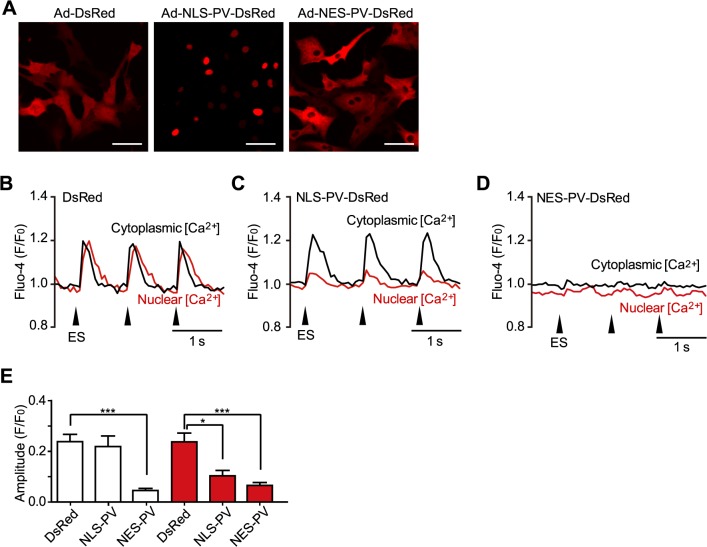
Effects of region-specific Ca^2+^ chelation on electrical stimulation-induced Ca^2+^ transients in cardiomyocytes. Confocal fluorescence images of NMVMs infected with adenoviruses encoding DsRed, nuclear-localized- or nuclear-excluded-parvalbumin (NLS-PV-DsRed or NES-PV-DsRed, respectively). Bars, 50 μm. (B–E) Representative traces (B-D) and summarized data (E) of cytoplasmic and nuclear Ca^2+^ transients elicited by 1-Hz electrical stimulation (ES) in NMVMs infected with Ad-DsRed as a control (B and E, n = 20), Ad- NLS-PV-DsRed (C and E, n = 8). *P < 0.05, vs. control; and Ad- NES-PV-DsRed (D and E, n = 9). *P < 0.05 and ***P < 0.001 vs. DsRed; values are expressed as means ± SEM.

To determine the source of nuclear Ca^2+^, we used a Ca^2+^ buffer protein, parvalbumin (PV), which selectively chelates Ca^2+^. We constructed adenoviruses encoding NLS-PV-DsRed, which is localized to the nucleus by a nuclear localizing signal (NLS), and NES-PV-DsRed, which is localized to the cytosol by a nuclear exclusion signal (NES), as well as the control DsRed. The localization of these proteins was verified by DsRed signals (Fig 2A). When the NMVMs were infected with Ad-DsRed, both cytoplasmic and nuclear Ca^2+^ transients were detected during electrical stimulation (Fig 2B). However, when the NMVMs were infected with NLS-PV-DsRed, which chelates nuclear Ca^2+^, increase of electrical stimulation-elicited nuclear Ca^2+^ transients was significantly attenuated, as expected, whereas cytoplasmic Ca^2+^ transients remained largely unaffected (Fig 2C and 2E). On the other hand, when cytoplasmic Ca^2+^ was chelated with NES-PV-DsRed, increases in both cytoplasmic and nuclear Ca^2+^ transients were abolished (Fig 2D and 2E). These results suggest that electrical stimulation-elicited nuclear Ca^2+^ transients are mostly derived from the cytoplasm.

### Effects of receptor stimulation on nuclear and cytoplasmic Ca^2+^ transients

**Fig 3:**
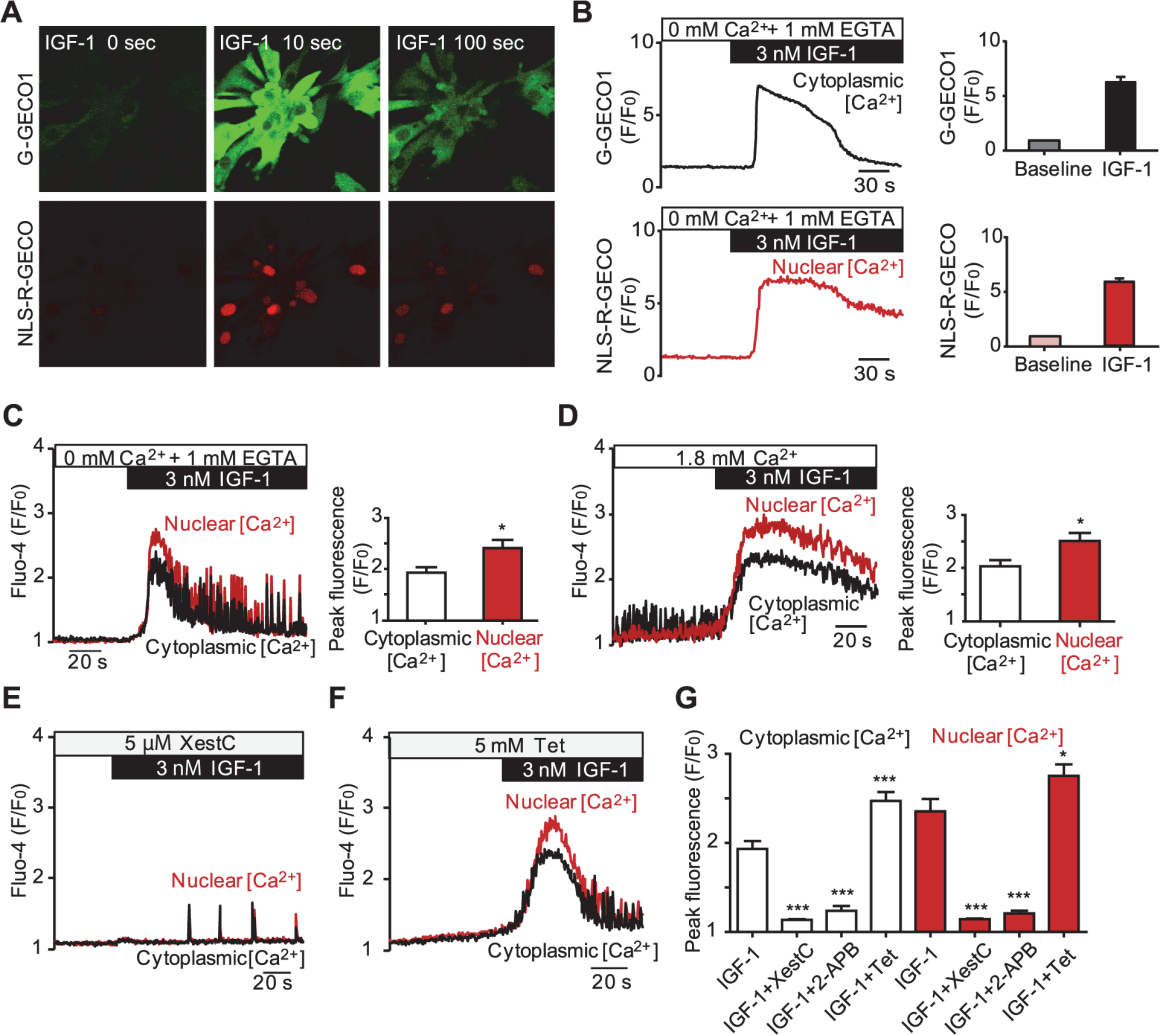
Effect of receptor stimulation on cytoplasmic and nuclear Ca^2+^ transient in cardiomyocytes. (A and B) Time-lapse images and representative traces from cultured NMVMs co-infected with adenoviruses encoding G-GECO1 and NLS-R-GECO before and after treatment with 3 nmol/L insulin-like growth factor 1 (IGF-1). Averaged data are shown in the bar graph. n = 10. (C and D) Effect of IGF-1 (3 nmol/L) on cytoplasmic and nuclear Ca^2+^ transients in NMVMs measured with Fluo-4 AM in Ca^2+^-free recording medium containing 1 mmol/L ethylene glycol tetraacetic acid (EGTA) (C) or in the presence of 1.8 mmol/L extracellular Ca^2+^ (D). Representative traces and averaged data of peak amplitude (n = 25 and 31, respectively, *P < 0.05 vs. cytoplasmic [Ca^2+^]). (E–G) Effects of inhibitors of IP_3_Rs (E) and RyRs (F) (5 μmol/L xestospongin C [XestC] and 5 mmol/L tetracaine [Tet], respectively) on the IGF-1-induced intracellular Ca^2+^ transients. (G) Summarized data of the peak levels of cytoplasmic and nuclear Ca^2+^ transients induced by IGF-1 with or without inhibitors. n = 16–22, *P < 0.05, ***P < 0.001 vs. IGF-1 only. Values are expressed as means ± SEM.

Next, we examined the effects of receptor stimulation on cytoplasmic and nuclear [Ca^2+^] in cultured NMVMs. We used insulin-like growth factor 1 (IGF-1) as an agonist of IP_3_R signals in these experiments. NMVMs infected with Ad-G-GECO1 and Ad-NLS-R-GECO were treated with 3 nmol/L IGF-1 under Ca^2+^-free conditions to exclude the effects of extracellular Ca^2+^. IGF-1 stimulation elicited large transient increases in the fluorescent signals of both G-GECO1 and NLS-R-GECO (Fig 3A), and their fluorescent intensity analysis clearly showed that IGF-1 induced large Ca^2+^ transients in both the cytoplasm and nucleus nearly simultaneously within the same cells (Fig 3B). Similar results were also obtained when we used Fluo-4 AM as the Ca^2+^ indicator (Fig 3C). However, the averaged data revealed that the peak amplitude of nuclear Ca^2+^ transients induced by IGF-1 was significantly higher than that of cytoplasmic Ca^2+^ transients (Fig 3C). Furthermore, larger nuclear Ca^2+^ transients compared to cytoplasmic Ca^2+^ transients were detected in the Ca^2+^-containing recording medium (Fig 3D). These results suggest that receptor stimulation preferentially triggers nuclear Ca^2+^ transients. The time to peak of the nuclear and cytoplasmic Ca^2+^ transients were similar (or the difference was undetectable) under our experimental conditions (data not shown). To clarify which Ca^2+^ release channels are involved in the IGF-1-induced Ca^2+^ transients, we examined the effects of IP_3_R and RyR inhibitors. In both the cytoplasm and nucleus, responses to IGF-1 were attenuated by preincubation with a specific IP_3_R inhibitor, xestospongin C (Xest C; Fig 3E and 3G) as well as by another IP_3_R inhibitor, 2-APB (Fig 3G). In contrast, tetracaine, a RyR inhibitor, was ineffective (Fig 3F and 3G). In addition to the above observation, we often detected that IGF-1-induced Ca^2+^ transients were accompanied by Ca^2+^ oscillations (e.g., during the decline phase of the Ca^2+^ transients in Fig 3C). These Ca^2+^ oscillations were also largely eliminated by Xest C treatment (Fig 3E), but not by tetracaine (Fig 3F). Taken together, these results demonstrate that IGF-1-induced cytoplasmic and nuclear [Ca^2+^] increases are mainly mediated by IP_3_Rs in mouse cardiomyocytes.

### Involvement of NCS-1 in IGF-1-induced nuclear and cytoplasmic Ca^2+^ transients in cardiomyocytes

**Fig 4:**
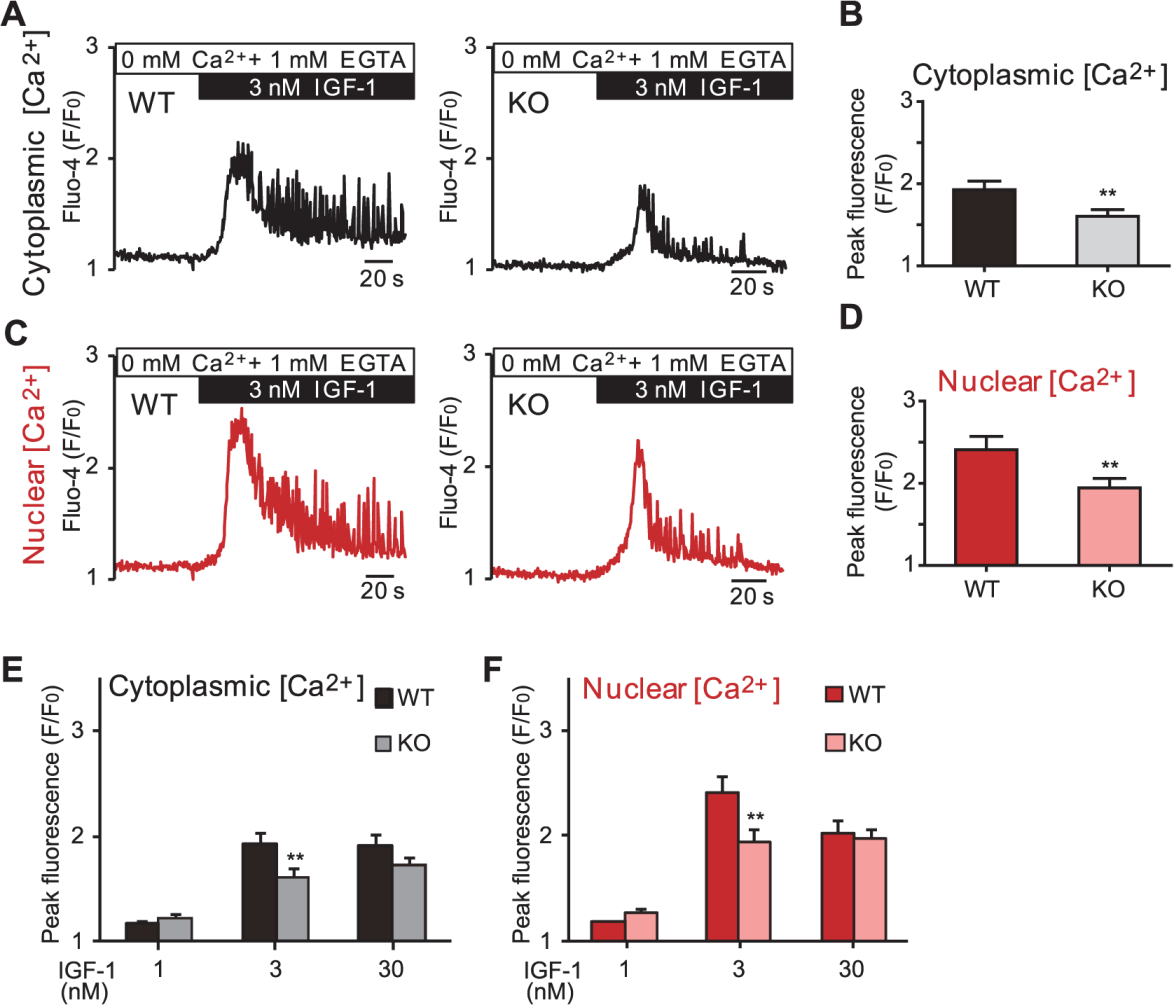
Involvement of neuronal calcium sensor-1 in IP_3_R-dependent Ca^2+^ release in cardiomyocytes. Comparison of the effects of IGF-1 stimulation on cytoplasmic and nuclear Ca^2+^ levels between wild type (WT) and neuronal calcium sensor-1 (NCS-1) deficient (KO) NMVMs. (A–D) Representative traces and averaged data of the peak fluorescence intensity (F/F_0_) of cytoplasmic and nuclear Ca^2+^ transients. n = 25 for WT and 37 for KO. (E and F) Summarized data obtained from the concentration-dependent experiments analyzing cytoplasmic and nuclear Ca^2+^ transients induced by 1, 3, and 30 nmol/L IGF-1. n = 11–30, **P < 0.01 vs. WT; values are expressed as means ± SEM.

We previously reported that IP_3_R-dependent Ca^2+^ signals are at least in part mediated by NCS-1 in mouse cardiomyocytes [11]. Therefore, in the present study, we investigated whether NCS-1 is also involved in nuclear Ca^2+^ signal regulation using NCS-1-deficient (KO) mouse cardiomyocytes. As described earlier (Fig 3C), stimulation of NMVMs with IGF-1 induced large cytoplasmic and nuclear Ca^2+^ transients accompanied by Ca^2+^ oscillations in wild type (WT) myocytes (Fig 4A and 4C, left panels). However, in KO myocytes, the effects of IGF-1 were significantly reduced in the cytoplasm (Fig 4A and 4B), similar to our previous observation using another IP_3_R signal agonist [11]. Interestingly, IGF-1-induced nuclear Ca^2+^ transients as well as Ca^2+^ oscillations were also significantly smaller in KO myocytes (Fig 4C and 4D). Concentration-dependent experiments for IGF-1 revealed that IGF-1 was most effective at the physiological range (3 nmol/L) in WT myocytes, and the Ca^2+^ transient amplitude was significantly reduced in KO myocytes in both the cytoplasm and nucleus (Fig 4E and 4F). These results strongly suggest that NCS-1 contributes to IGF-1-induced nuclear (as well as cytoplasmic) Ca^2+^ regulation, possibly through IP_3_Rs, in mouse cardiomyocytes.

### Co-localization of NCS-1 and IP_3_Rs in mouse cardiomyocytes

**Fig 5:**
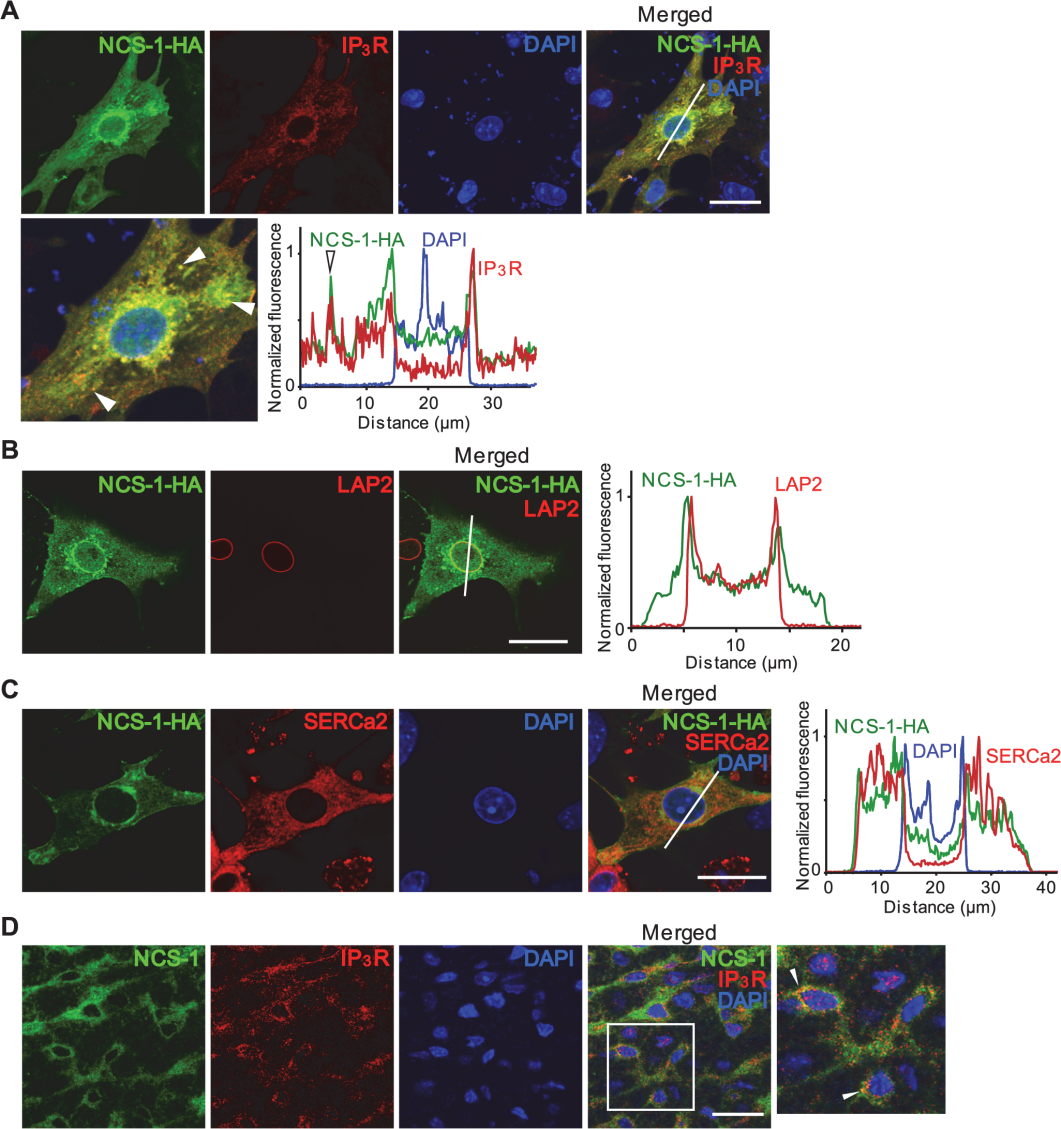
Similar localization pattern of NCS-1 and IP_3_Rs in cardiomyocytes. (A–C) Confocal images showing the subcellular localization pattern of NCS-1 together with IP_3_Rs (A), lamina-associated protein 2 (LAP2), an inner nuclear membrane protein (B), or SERCa2 (C), in NMVMs overexpressing NCS-1-HA. Bars, 20 μm. Linear fluorescence profile analysis of the white lines in the images are shown in the right panel. In panel A, white arrowheads indicate co-localization of NCS-1-HA and IP_3_Rs in the cytosol. (D) Confocal images showing localization of endogenous NCS-1 and IP_3_Rs in the left ventricular myocardium obtained from a 1-week old mouse. White arrowheads indicate co-localization between NCS-1 and IP_3_Rs. Bar, 20 μm. Nuclei were counterstained with DAPI. Bar, 20 μm. Three independent experiments were performed.

Immunocytochemical analysis revealed that NCS-1 and IP_3_Rs were widely co-localized within NMVMs overexpressing NCS-1-HA, especially around the nucleus [11] and in the cytosol with a punctate distribution (Fig 5A). Counterstaining with lamina-associated protein-2 (LAP2), an inner membrane protein of the nuclear envelope, revealed that some NCS-1 is localized in the perinuclear region, but probably not within the nucleus, because the peaks of NCS-1 and LAP2 were not superimposed in the linear fluorescence profile analysis (Fig 5B). In addition, some NCS-1 was co-localized with an SR marker, SERCa2 (Fig 5C), suggesting that NCS-1 is present in the SR, where IP_3_Rs are also present. Such co-localization between NCS-1 and IP_3_Rs was observed for endogenous NCS-1 expressed in native myocardium obtained from 1-week-old mouse left ventricle (Fig 5D). These results indicate that NCS-1 is co-localized with IP_3_Rs in the perinuclear and other subcellular regions, including the SR, in mouse cardiomyocytes.

### IGF-1 enhances the interaction between NCS-1 and IP_3_Rs in cardiomyocytes

**Fig 6:**
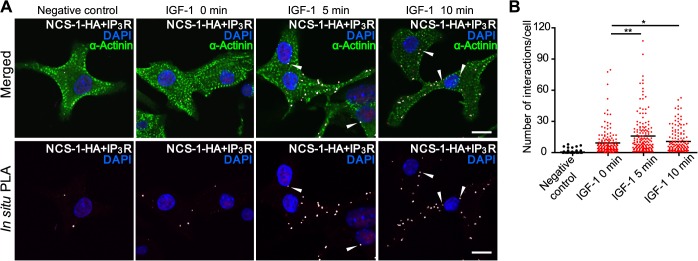
Effects of IGF-1 stimulation on the interaction between NCS-1 and IP_3_Rs in cardiomyocytes. (A and B) Interactions between NCS-1 and IP_3_Rs were visualized and quantified using *in situ* proximity ligation assay (PLA) in α-actinin-positive NMVMs expressing NCS-1-HA. (A) Representative *in situ* PLA images of NCS-1-HA/IP_3_R interactions (detected as white dots) before and 5 or 10 min after IGF-1 (10 nmol/L) treatment. α-Actinin-positive cells (myocytes) were stained green, and nuclei were stained blue by DAPI. White arrowheads indicate the signals with close proximity to the nuclear membrane. Experiments were also carried out without primary antibodies as a negative control (leftmost). Bar, 10 μm. (B) Quantification of the NCS-1-HA/IP_3_R interactions. The number of interactions/cell was plotted for all the cells and the mean values for each group are presented as bars. *P < 0.05, **P < 0.01 vs. IGF-1 0 min; n = 80–88 in three independent experiments.

We next investigated whether IGF-1 stimulation alters the interaction between NCS-1 and IP_3_R. We performed *in situ* PLA to examine the interaction between NCS-1 and IP_3_R. In basal conditions, only a few punctate fluorescent signals (indicative of physical interaction between NCS-1 and IP_3_Rs) were detected in the α-actinin-positive NMVMs (Fig 6A, second panel from the left). However, IGF-1 treatment significantly increased the number of fluorescent signals (Fig 6A, third and fourth panels from the left). The number of interactions per cell significantly increased following 5- and 10-min IGF-1 treatments (Fig 6A and 6B). Several PLA signals were detected around the nuclei (Fig 6A, arrowheads); however, majority of the signals were detected in other regions of cells. Very few PLA signals were detected in the negative control, to which no primary antibodies were added (Fig 6A, the leftmost panels). These results demonstrate that IGF-1 stimulation increases physical interaction between NCS-1 and IP_3_Rs and suggest that such interaction may extend the function of IP_3_Rs, thereby increasing nuclear [Ca^2+^] in cardiomyocytes.

## Discussion

### Ca^2+^ indicators for distinctive measurement of cytoplasmic and nuclear [Ca^2+^] in cardiomyocytes

We focused on distinctive measurement of intracellular [Ca^2+^] in various subcellular compartments. With the recently developed genetically encoded fluorescent Ca^2+^ probes, we can simultaneously detect region-specific [Ca^2+^] changes, whereas with conventional Ca^2+^ indicator, we can quantitatively compare the [Ca^2+^] between the different regions. Using GECOs, we confirmed that nuclear Ca^2+^ transients were indeed elicited by both electrical and receptor stimulations in NMVMs. Because GECO has not been used previously for Ca^2+^ measurements in cardiomyocytes, our results also provide an evaluation of this Ca^2+^ indicator. We were able to detect a large receptor stimulation-elicited Ca^2+^ transient in GECO-expressing myocytes; conversely, the signal/noise ratio of electrical stimulation-elicited Ca^2+^ transients in the NLS-R-GECO-expressing cells was small (see Fig 1), although all Ca^2+^ transients were measured clearly with Fluo-4. This is possibly due to the lower dynamic range and dissociation rate of GECOs than those of Fluo-4 [21]. Thus, GECOs may be more suitable for measuring minute-scale reactions such as receptor stimulation-elicited Ca^2+^ changes, rather than for very rapid changes in [Ca^2+^], such as those elicited by electrical stimulation.

### Distinct mechanisms of nuclear [Ca^2+^] regulation in response to electrical and receptor stimulations

**Fig 7:**
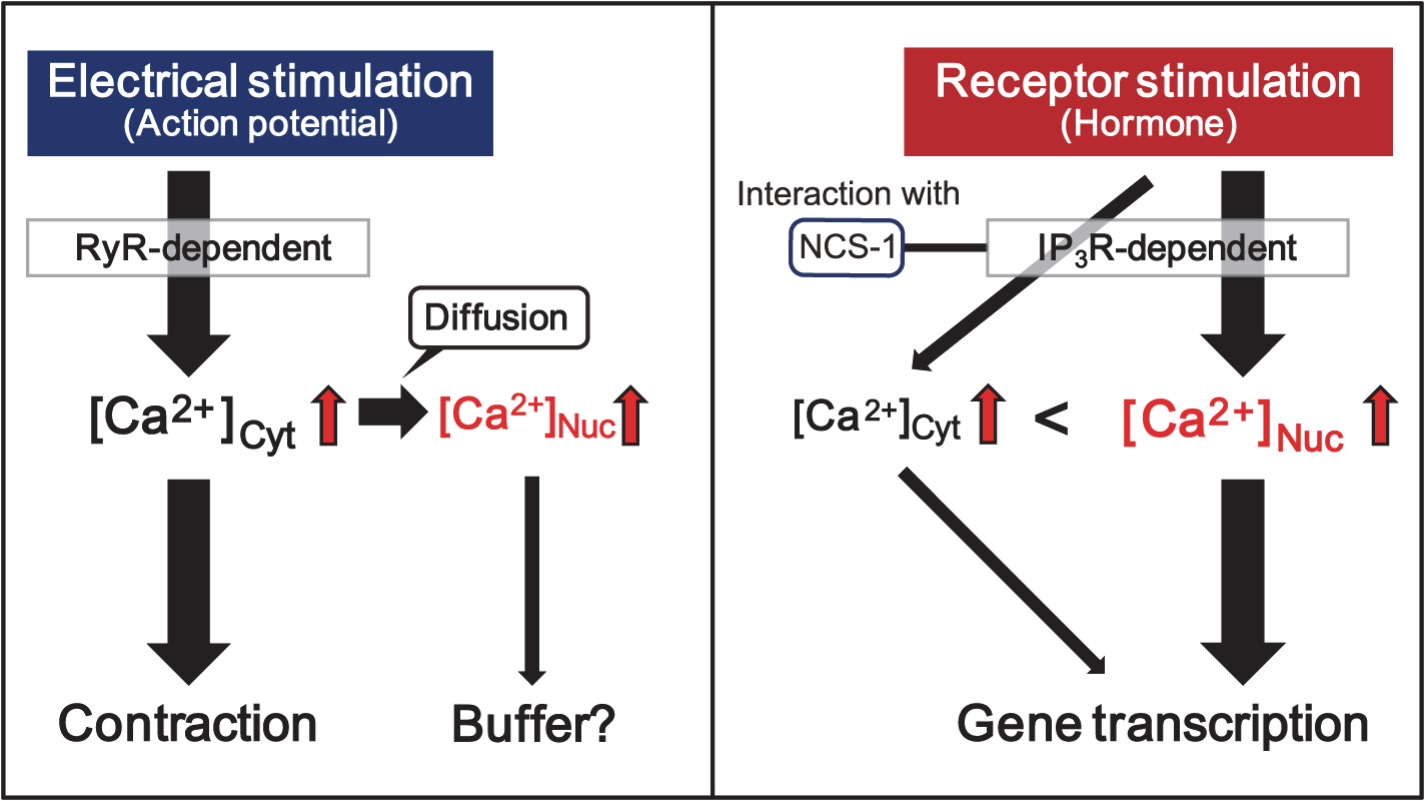
Possible mechanism for cytoplasmic and nuclear [Ca^2+^] regulation in response to different stimuli in cardiomyocytes. During electrical stimulation, nuclear [Ca^2+^] ([Ca^2+^]_Nuc_) is elevated by influx of increased cytoplasmic [Ca^2+^] ([Ca^2+^]_Cyt_), which is released from the sarcoplasmic reticulum (SR) via an RyR-dependent mechanism called Ca^2+^-induced Ca^2+^ release. Elevated [Ca^2+^]_Cyt_ results in muscle contraction, and the nucleus may buffer excessively increased [Ca^2+^]_Cyt_. On the other hand, receptor stimulation preferentially increases [Ca^2+^]_Nuc_ rather than [Ca^2+^]_Cyt_, which may be mainly mediated by IP_3_Rs. Receptor stimulation-induced increase in intracellular [Ca^2+^] may enhance gene transcription, leading to cardiac hypertrophy. NCS-1 plays important roles in receptor stimulation-mediated nuclear [Ca^2+^] regulation through interaction with IP_3_Rs in mouse cardiomyocytes.

In electrically stimulated myocytes, we observed that nuclear Ca^2+^ transients followed cytoplasmic Ca^2+^ transients; they were eliminated upon treatment with RyR inhibitor; and largely inhibited by chelation of cytoplasmic Ca^2+^ with parvalbumin. These results suggest that the Ca^2+^ source for the nuclear Ca^2+^ transients cytoplasmic. In this scenario, electrical stimulation first increases cytoplasmic [Ca^2+^] by a RyR-mediated “Ca^2+^-induced Ca^2+^ release” mechanism, and subsequently, some of these Ca^2+^ ions might be transferred to the nucleus possibly through nuclear pore complexes, accounting for the majority of nuclear Ca^2+^ transients. Elevated cytoplasmic [Ca^2+^] results in muscle contraction, and the nuclei may function as Ca^2+^ buffering stores for excessive cytoplasmic Ca^2+^ ions during E-C coupling (Fig 7). However, the slower T_1/2_ of decline indicates that the Ca^2+^-extrusion mechanism in the nucleus may be different from that in the cytoplasm. In the cytosol, increased [Ca^2+^] rapidly declines upon activation of the plasma membrane- and SR-Ca^2+^ pumps, as well as the Na^+^-Ca^2+^ exchanger. On the other hand, the presence of Ca^2+^ pumps on the inner membrane of the nuclear envelope has not been verified (although, Na^+^-Ca^2+^ exchanger is present on the nuclear envelope in neurons [23]). Therefore, passive diffusion through nuclear pore complexes probably accounts for the decline of nuclear [Ca^2+^], and this may account for the slower kinetics of Ca^2+^ decline in the nucleus when compared with that in the cytoplasm.

On the other hand, receptor stimulation with IGF-1 preferentially increases nuclear [Ca^2+^] (both Ca^2+^ transients and Ca^2+^ oscillations) rather than cytoplasmic [Ca^2+^], and this response is largely mediated by IP_3_Rs (Fig 3), similar to the results obtained with other IP_3_R agonists such as endothelin-1 and IP_3_ in cardiomyocytes [3,4,6]. Ca^2+^ oscillations are thought to arise due to periodic release of Ca^2+^ from intracellular Ca^2+^ stores via IP_3_R [24]. That is, IP_3_R is activated at low cytosolic [Ca^2+^] [25], whereas it is inhibited at high [Ca^2+^] [26]. Therefore, in vivo, the binding of IP_3_ together with fluctuating cytosolic [Ca^2+^] can trigger successive cycles of IP_3_R activation and inhibition, which result in Ca^2+^ oscillations. Each size of monophasic Ca^2+^ transients and Ca^2+^ oscillations is determined by the localization of different subtype of IP_3_Rs [27]. Preferential elevation of nuclear [Ca^2+^] by receptor stimulation may be attributed to the fact that IP_3_Rs are largely expressed in the inner and outer membranes of the nuclear envelope [9,28], which might function as exclusive Ca^2+^ stores for the nucleus. Because IP_3_-sensitive release of Ca^2+^ from perinuclear Ca^2+^ stores is reported to activate two Ca^2+^-dependent hypertrophic pathways (calcineurin/nuclear factor of activated T-cells (NFAT) [8] and the Ca^2+^/calmodulin-dependent protein kinase II (CaMKII)/histone deacetylase (HDAC) pathways [3]), receptor stimulation-elicited [Ca^2+^] elevation in the nucleus observed in this study would contribute to the gene transcription program that precedes cardiac hypertrophy (Fig 7)

### NCS-1 mediates nuclear [Ca^2+^] regulation and its potential mechanisms

We observed that IGF-1-induced nuclear Ca^2+^ transients and Ca^2+^ oscillations were significantly smaller in NCS-1 KO NMVMs, compared with WT cells (Fig 4), indicating that NCS-1 is involved in the IP_3_R-dependent nuclear Ca^2+^ release. On the basis of our results, we propose three possible mechanisms for NCS-1-mediated nuclear [Ca^2+^] regulation. First, there might be direct activation of Ca^2+^ release into the nucleoplasm via NCS-1-dependent acceleration of intra-nuclear IP_3_R activity. Second, there may be an indirect effect of increased Ca^2+^ diffusion through the nuclear pore complexes via activation of perinuclear (extra-nuclear) IP_3_R by interaction with NCS-1. Third, NCS-1 may maintain Ca^2+^ contents in the nuclear envelope by increasing the SR Ca^2+^ content, thereby increasing IP_3_R-mediated Ca^2+^ release into nucleoplasm.

Nuclear IP_3_ binds to IP_3_Rs located in the inner membrane of the nuclear envelope, leading to Ca^2+^ release into the nucleoplasm [3–6]. Immunofluorescence analyses have demonstrated that NCS-1 and IP_3_Rs are co-localized around the nucleus (Fig 5A). NCS-1 is a small molecule (~22 kDa) that, like IP_3_, would pass through the nuclear pore complexes easily. These results support our first hypothesis that NCS-1 directly regulates IP_3_Rs from inside the nucleus. However, the expression of endogenous NCS-1 inside the nucleus appears to be very low in cardiomyocytes (Fig 5D). In addition, fractionation analysis revealed that NCS-1 is strongly expressed in the cytosolic and membrane fractions, but only slightly expressed in the nuclear fraction (data not shown). These results suggest that NCS-1 only contributes to a small extent to the function of IP_3_Rs expressed in the inner membrane of the nuclear envelope.

There is an emerging concept in Ca^2+^ signaling that supports another scenario—increased diffusion of Ca^2+^ through the nuclear pore complexes. Ca^2+^ signals that arise in the perinuclear region in response to receptor stimulation with IP_3_R agonists can simultaneously elevate [Ca^2+^] within the cytoplasm and nucleoplasm in cardiomyocytes [3,4,8]. Ca^2+^ diffuses more slowly in the cytosol than in the nucleoplasm because of greater buffering capacity in the cytosolic compartments [29]. Therefore, localized perinuclear Ca^2+^ release into the cytoplasm is predominantly followed by diffusion of Ca^2+^ through the nuclear pore complexes into the nucleoplasm, and this can affect nuclear activities. Our immunofluorescence analysis clearly demonstrated that NCS-1 is expressed just outside the nucleus (Fig 5B), and it is co-localized with the IP_3_Rs, particularly in the perinuclear region of cardiomyocytes (Fig 5A and 5D). Therefore, we propose that extra-nuclear NCS-1 contributes significantly to IP_3_R-dependent nuclear [Ca^2+^] regulation.

Another possible mechanism of NCS-1 action is that it might increase the Ca^2+^ content of the nuclear envelope. We previously reported that NCS-1 increases the SR Ca^2+^ content via activation of IP_3_Rs followed by activation of CaMKII and SR Ca^2+^ pump activity in cardiomyocytes [11]. Moreover, SR and the nuclear envelope are highly interconnected Ca^2+^ stores [30]. Therefore, it is possible that NCS-1 increases the Ca^2+^ content of nuclear envelope through activation of IP_3_Rs on the SR. Consistent with this hypothesis, *in situ* PLA analysis revealed that the IGF-1-induced increase in the interaction between NCS-1 and IP_3_Rs was largely detected in the subcellular regions distal from the nucleus (Fig 6A). Some of these regions might correspond to the SR, because immunofluorescence analysis clearly demonstrated that NCS-1 is present in this location (Fig 5C), where IP_3_Rs are also present. We indeed observed that NCS-1 and IP_3_Rs were co-localized not only at the perinuclear regions but also in punctate cytosolic regions (Fig 5A, arrowheads in the enlarged photograph); this is consistent with the reported localization pattern of IP_3_Rs [27]. Thus, NCS-1 may function in maintaining sufficient [Ca^2+^] in the nuclear envelope, leading to Ca^2+^ release from the nuclear envelope into the nucleoplasm via IP_3_Rs expressed in the inner membrane of the nuclear envelope. Further studies are required to discriminate the predominant mechanism that underlies NCS-1-mediated enhancement of nuclear Ca^2+^ signaling.

## Conclusion

Using two different kinds of Ca^2+^ indicators, we performed simultaneous measurements of nuclear and cytoplasmic [Ca^2+^] in cardiomyocytes. Our results suggest that distinct mechanisms exist for the regulation of nuclear [Ca^2+^] in response to electrical and receptor stimulations in the heart. Moreover, our results revealed a novel role for NCS-1 in the regulation of nuclear [Ca^2+^], mediated by IP_3_Rs in mouse cardiomyocytes. Receptor stimulation with IGF-1, enhances the interaction between NCS-1 and IP_3_Rs, and promotes nuclear [Ca^2+^] elevation. We have previously demonstrated that phenylephrine-induced cardiac hypertrophy was significantly attenuated in NCS-1 KO hearts concomitant with a reduced activation of both calcineurin/NFAT and CaMKII/HDAC pathways [11]. Therefore, NCS-1-dependent nuclear [Ca^2+^] regulation may also be involved in cardiac hypertrophy through these pathways.
